# WeChat as a Platform for Problem-Based Learning Among Hematological Postgraduates: Feasibility and Acceptability Study

**DOI:** 10.2196/16463

**Published:** 2021-05-25

**Authors:** Ping Luo, Wenwen Pang, Yingying Wang, Minghui Liu, Shu Zhou, Shuai Liu, Xian Zhang, Li Liu, Yanan Liu, Fuling Zhou

**Affiliations:** 1 Department of Hematology Zhongnan Hospital of Wuhan University Wuhan China; 2 Department of Hematology Dawu County People’s Hospital Xiaogan China

**Keywords:** problem-based learning, PBL, WeChat, hematology, postgraduate, education

## Abstract

**Background:**

Hematological medicine is a practical discipline that is difficult to study. Problem-based learning (PBL) is an innovative student-centered teaching method wherein students define their own learning objectives from clinically based problems. Considering that WeChat is the most popular communication app in China, we selected it as a new platform for online PBL to reduce the limitations of traditional PBL in hematology teaching.

**Objective:**

This study aims to explore a new pedagogical method called WeChat-PBL, which is based on real micro clinical cases for postgraduates majoring in hematology and to demonstrate its feasibility and acceptability.

**Methods:**

A total of 48 hematological postgraduates and 7 tutors participated in this study. We divided the participants into 7 groups where students can learn theoretical knowledge. After each course, the members of each group were required to complete in-class quizzes. Moreover, the students and tutors were required to fill out periodic (after each class) and overall (after each semester) evaluations.

**Results:**

A total of 8 micro clinical cases were presented in WeChat-PBL. The average quiz score for acute myelogenous leukemia, chronic myeloid leukemia, multiple myeloma, acute promyelocytic leukemia, and lymphoma were 89.0%, 86.0%, 83.4%, 88.8%, and 77.5%, respectively. Periodic evaluations showed that both students and tutors were satisfied with the process of WeChat-PBL. The overall evaluation results showed that WeChat-PBL was able to positively impact the learning experiences of hematological postgraduates.

**Conclusions:**

Our results indicate the feasibility and acceptability of the WeChat-PBL teaching method for postgraduates majoring in hematology.

## Introduction

Postgraduate education is an important way to expand the knowledge and skills of students [1]. Today, with the continuous advancement of teaching reforms in China, the number of medical students enrolled in postgraduate degrees is increasing, especially in hematology. Hematology is a branch of medicine that explores the diagnosis, treatment, and prevention of diseases related to blood. The clinical manifestations of patients with hematological diseases are often serious and progress rapidly [2]. Both the diagnosis and treatment of hematological diseases are complex. For example, the diagnosis of leukemia, a serious disease in the hematological system, requires a number of laboratory examinations, including blood, bone marrow, immunity, molecular, and genetic analyses, which makes it difficult for students to master knowledge on blood diseases. All of these characteristics make hematology a difficult subject to learn, and students need to change their role from passive to active. The choice of appropriate teaching methods is also important. Problem-based learning (PBL) may be one of the best methods for teaching hematology postgraduates.

The traditional pedagogy, which is teacher centered, class oriented, and examination driven, places students in a passive state of “reception” [3]. PBL is a student-centered instructional method wherein students define their own learning objectives from clinically based problems [4,5]. As an established approach, PBL has been reported to be suitable for use in graduate entry medical schools [6]. Recently, PBL has become the subject of considerable interest in postgraduate education [7]. PBL can not only cultivate postgraduates’ leadership, teamwork, communication, and problem-solving abilities, which are useful for lifelong learning, but also facilitate postgraduates to become responsible for their own learning. Mindaugas and colleagues [8] found that PBL may be an effective approach for postgraduates because the PBL learning process is more demanding and self-directed, which facilitates independent and creative thinking. However, we must also admit that traditional PBL has its limitations. Generally, PBL students must get together in the classroom to discuss and share opinions. Considering the geographic and time dispersion of students, traditional PBL among hematological postgraduates is difficult to conduct. Therefore, the traditional PBL should be modified to address the physical and temporal restrictions in this cohort.

With the development of the internet in China, traditional PBL can be feasibly transferred to an online PBL setting in a virtual environment. The new mode of PBL would be constructed using modern digital technology. Thus, the time and physical restrictions of traditional PBL education would be eliminated. A previous study validated digital PBL, stating that it is as effective as traditional PBL and more effective than traditional learning in improving knowledge [9]. WeChat is one of the fastest growing mobile apps developed by Tencent, with over 697 million currently active user accounts [10], and it is the most popular platform among university students in China [11]. It has connected more than half a billion Chinese people at present. Similar to WhatsApp, WeChat permits users to send messages to an individual or a specific group in various formats, including texts, videos, voice recordings, and images [12]. WhatsApp has been introduced into medical education in colleges and universities in Western countries with great success [13,14]. In China, WeChat has also become increasingly popular as an interactive communication tool in medical education [15]. Thus , WeChat may be an appropriate tool for online PBL, which can eliminate the physical limitations of traditional PBL.

This study aims to explore a new pedagogical method called WeChat-PBL, which is based on real micro clinical cases for postgraduates majoring in hematology, and to demonstrate its feasibility and acceptability.

## Methods

### Participants

A total of 48 hematological postgraduates studying in Zhongnan Hospital, Wuhan University, were enrolled in our study. Clinical doctors (n=7) with 1 or more years of experience in traditional PBL teaching were assigned as tutors in the WeChat-PBL groups. Our new mode of PBL was constructed on the basis of the WeChat app. WeChat is a popular app that is available on Android, iPhone, and Windows. It is supported by Wi-Fi, 4G, and 5G data networks. Using WeChat, students can communicate with each other anywhere and at any time [16]. All students who participated in this project had their own mobile phones with the WeChat app installed. All were familiar with the practical aspects of WeChat in the PBL context. We randomly divided the participants into 7 groups. Each group comprised 6 or 7 students and 1 tutor. WeChat-PBL was used from August to October 2020. Informed consent was provided to the participants and signed prior to beginning the study. The study was approved by the Medical Council of Wuhan University.

### The WeChat-PBL Pedagogy Method

Micro clinical cases including clinical characteristics and relevant questions were uploaded to the WeChat-PBL group 3 days before class. The WeChat group included a tutor and 6 or 7 postgraduate students. Then, the 7 members were divided into 2 groups to create their own Microsoft PowerPoint (Microsoft Corp) according to the micro medical cases the teacher presented by reading books, reviewing the literature, or asking others. In the PBL class, the representative of each group uploaded their PowerPoint and showed it to the others using the video conferencing function in WeChat. Members in the WeChat-PBL group could then pose questions about the selected case and discuss various issues by sending text, images, voice recordings, videos, or documents. As a person who facilitates and motivates learning in the group, the tutor summarized and explained the questions. Owing to the functions of WeChat, all the messages could be easily and instantly read, making WeChat-PBL highly efficient.

### The WeChat-PBL Student Examination System

PBL is a learner-centered instructional method by which students learn content and thinking strategies [17]. Self-regulated learning skills play an important role in learning efficiency [18]. Some students are highly self-disciplined, whereas others are not. To help students with poor self-discipline, examination is necessary. All participants were required to complete a quiz after each class, graded by the tutor, and the average score was calculated based on the quiz result. The tutor visually displayed the students’ progress through the WeChat-PBL group and monitored students who performed poorly. This assessment was conducted immediately after each class.

### Evaluating WeChat-PBL

**Figure 1:**
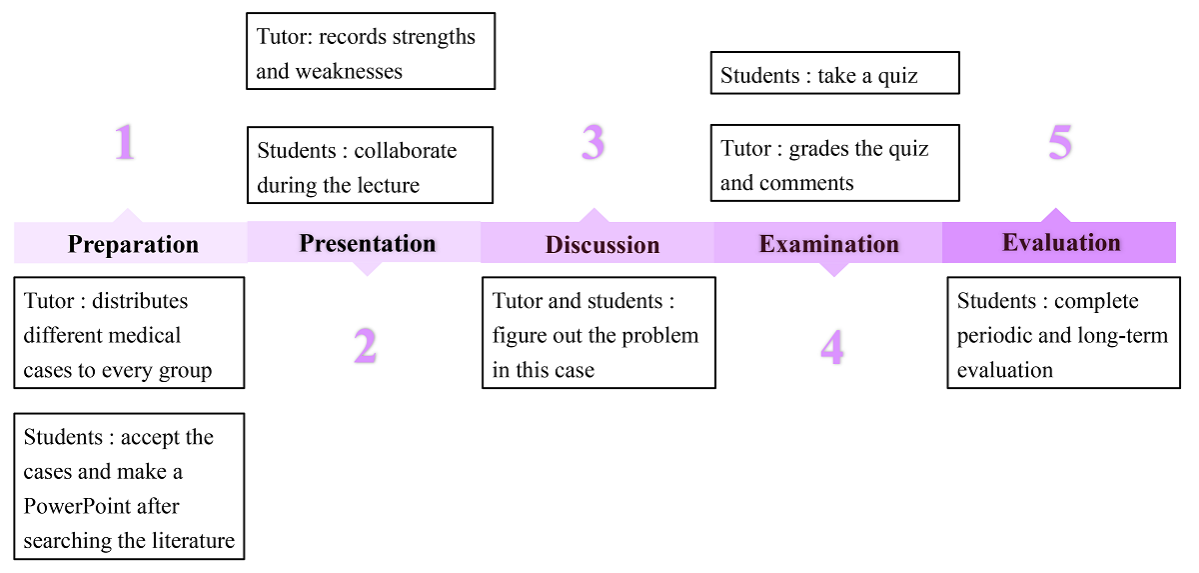
Basic flowchart of the WeChat-PBL teaching mode.

Curriculum evaluation is important to ensure the quality of education [19], and this also applies to WeChat-PBL. The key to evaluating WeChat-PBL is to assess whether postgraduates are effectively gaining knowledge, improving skills, and developing scientific research abilities. The evaluations were divided into 2 sections: periodic evaluation of the WeChat-PBL process and an overall evaluation of the whole WeChat-PBL project. The students and tutors were required to complete the periodic evaluation after each PBL class (eg, evaluation of students’ participation in creating PowerPoints and performance in the WeChat-PBL group). The overall evaluation of WeChat-PBL was conducted in the form of a group discussion after each semester. The questions were designed in accordance with previous studies [20,21] and with the specific features of this study. The questions displayed in the *Results* section were discussed by all tutors who participated in this research to ensure its quality. The basic flowchart of the WeChat-PBL mode is shown in Figure 1.

### Analysis

Microsoft Excel 2013 (Microsoft Corp) was used to perform the data analysis. The average score for each micro clinical case (ie, disease) was calculated by adding up all the quiz scores and divided by the total number of participants involved in the case. Percentages were used to describe the overall evaluation results and was calculated by the number of participants who agreed with each item divided by the total number of participants (N=48). The content of the periodic evaluations within the WeChat-PBL groups was analyzed qualitatively.

## Results

### PBL Cases

In our study, 8 real clinical cases were presented in WeChat-PBL. A total of 5 different kinds of hematological diseases—acute myelogenous leukemia, chronic myeloid leukemia, multiple myeloma, acute promyelocytic leukemia, and lymphoma—were studied. Every WeChat-PBL group comprised 3 classes. It took 6-10 days to finish 1 PBL case.

### Student Examination

The average quiz score for the 5 kinds of hematological diseases were as follows: acute myelogenous leukemia, 89.0%; chronic myeloid leukemia, 86.0%; multiple myeloma, 83.4%; acute promyelocytic leukemia, 88.8%; and lymphoma, 77.5%. The majority of students (41/48, 85.4%) communicated actively within the groups, whereas 7 communicated less actively in the beginning. The tutor spoke with them privately, inquired about the reason behind the reduced participation, and encouraged them to actively take part in the discussions. Finally, all students became active in the group discussions.

### Evaluation of WeChat-PBL

Periodic evaluation results showed that every students played an active part in making their PowerPoint. The tutors were satisfied with the performance of the students and reported that students exhibited obvious improvement in both theoretical study and scientific research abilities.

The overall evaluation was conducted in the form of a group discussion. The results of this discussion are presented in Table 1. Most students had a better understanding of PBL (41/48, 85.4%). Almost all students were satisfied with their performance in WeChat-PBL, including completing the tasks assigned to them on time (44/48, 91.7%), applying prior knowledge to solve problems (48/48, 100.0%), and communicating ideas with group members effectively (42/48, 87.5%) The majority (44/48, 91.7%) believed that their clinical skills and scientific research abilities were improved through the use of WeChat-PBL. In addition, most of the students reported that group members and the tutor offered timely feedback (45/48, 93.8%) and held the view that the WeChat-PBL teaching mode was reasonable (42/48, 87.5%) and better than traditional PBL (39/48, 81.2%). The majority of students thought that WeChat was an effective app for online PBL for postgraduates majoring in hematology (39/48, 81.2%). In terms of the quality of the micro clinical cases presented in WeChat-PBL, the majority of students thought that it was valuable and interesting (43/48, 89.6%). Not surprisingly, almost all students felt that the role of the tutor was very important in the PBL group setting (46/48, 95.8%). However, only 30 out of 48 participants (62.5%) felt free to pose questions in the WeChat-PBL group.

**Table 1:** Results of the overall evaluation of the WeChat-PBL teaching mode.

Number	Question	Respondents, n (%)	
		Agree	Disagree
1	Did you have a better understanding of PBL^a^?	41 (85.4)	7 (14.6)
2	Did you enjoy the discussions in WeChat-PBL?	41 (85.4)	7 (14.6)
3	Did you complete the tasks assigned to you on time?	44 (91.7)	4 (8.3)
4	Did you apply prior knowledge to solve problems?	48 (100.0)	0 (0.0)
5	Did you feel free to pose questions?	30 (62.5)	18 (37.5)
6	Did you actively take part in the discussions?	34 (70.8)	14 (29.2)
7	Were you able to communicate ideas with your group members effectively?	42 (87.5)	6 (12.5)
8	Do you think that the micro clinical cases presented in WeChat-PBL are valuable and interesting?	43 (89.6)	5 (10.4)
9	Did your clinical skills and scientific research abilities improve through WeChat-PBL?	44 (91.7)	4 (8.3)
10	Did your group members and tutor offer timely feedback?	45 (93.8)	3 (6.2)
11	Do you think the teaching mode of WeChat-PBL is reasonable?	42 (87.5)	6 (12.5)
12	Do you think the teaching mode of WeChat-PBL is better than that of traditional PBL?	39 (81.2)	9 (18.8)
13	Did your tutor consistently show enthusiasm with PBL?	48 (100.0)	0 (0.0)
14	Do you think that the tutor played an important role in student learning in the PBL group?	46 (95.8)	2 (4.2)
15	Do you think that WeChat is an effective app for online PBL for postgraduates majoring in hematology?	39 (81.2)	9 (18.8)

^a^PBL: problem-based learning.

## Discussion

### Principal Findings

The new PBL mode based on WeChat was designed to promote the abilities of hematological postgraduates, including clinical reasoning, team skills, and metacognition. This mode of online PBL successfully eliminated the physical and temporal limitations of traditional PBL in postgraduate teaching. In our study, the average quiz score for acute myelogenous leukemia, chronic myeloid leukemia, multiple myeloma, and acute promyelocytic leukemia was very high. The majority of students reported that their clinical skills and scientific research abilities improved through WeChat-PBL and thought that WeChat was an effective app for online PBL for postgraduates majoring in hematology, indicating the acceptance and effectiveness of the WeChat-PBL teaching mode.

In this study, almost all students felt that the role of the tutor was very important in the PBL group. A previous study has also validated that the tutor plays an important role in student learning in the PBL group [22]. Dissatisfaction with PBL has arisen owing to the teaching capabilities and enthusiasm of the facilitators [23,24]. Barrows and colleagues [25] expressed the view that the tutor should facilitate and motivate learning rather than serve as a source of knowledge. However, other studies support the idea that the best tutors are those with both the ability to promote learning and present clinical content [26]. Dolmans et al [27] have reported that the tutor’s acts not only have an impact on the productiveness but also the effectiveness of the PBL group’s work. To help tutors become more effective in PBL tutorials, continuous training that can facilitate tutors’ reflections of their own development as teachers [28,29] and assist them in shifting from a traditional lecturing role to a multifaceted role as a mentor, coach, model, and guide [30] is needed.

The majority of data on tutor training has mainly focused on general moderation techniques for tutors [31]. In 2005, Azer [32] presented a group of challenges faced by PBL tutors and 12 tips for successful group facilitation. Tutors can read books, educational reviews, and research articles and regularly record teaching experiences by using reflective journals to achieve the essence of these tips in teaching. Moreover, PBL has 7 sequential steps, including case presentation, problem definition, brainstorming, generating hypotheses, defining learning goals, self-study, and synthesis [21]. Tutors should cultivate themselves according to these steps.

Eight micro clinical cases were presented in WeChat-PBL, and 89.6% of students found these cases to be valuable and interesting. Because the cases are brief and interesting, WeChat-PBL is time-efficient, effective, and interesting to the learner and teacher. Our study involved real-life cases and the actual experiences of health professionals, which is helpful to enhance the relevance of the subject matter. Professional knowledge is integrated with clinical presentations. Student learning, therefore, is associated with real-life situations.

PBL is a learner-centered instructional pedagogy, in which students play a central role in their learning [17]. Thus, several competencies including knowledge acquisition, practical skills, and professional attitudes are important for them to become active, cooperative, and self-directed [33]. Assessment of competence is vital for students to identify and respond to their own learning needs, providing insights into their actual performance. Examination drives learning, and it affects not only what students learn but also how they learn [34]. In our study, we assessed the students by evaluating their performance in the WeChat-PBL group, completion of the work, and a quiz after each class. We observed an obvious correlation between the examination scores of students who participated more actively than others.These data highlight the importance of effective assessment systems for monitoring student progress in the PBL curricula.

Of note, our WeChat-PBL teaching mode has several obvious advantages compared with the traditional PBL. First, WeChat is the most popular app in China [35]. It is a very convenient way for students to communicate. By uniting traditional classroom education with the WeChat app, students can learn actively at any time, both before and after class. Second, the frequent notifications in the WeChat group establish periodic contact between students, which encouraged student feedback on the course and facilitated student-student and student-teacher interactions. Third, more teamwork can be encouraged through WeChat-PBL than traditional PBL given that students can communicate easily with the help of the WeChat app. Fourth, the micro clinical cases were uploaded to the WeChat-PBL group before class. Students were able to prepare for the session in advance, thus cultivating the learner in an efficient, goal-directed manner.

### Conclusion

A great number of changes have taken place in the instructional methods used to teach medical students. Hematological medicine is a practical discipline that is difficult to master. In our study, we demonstrated the feasibility and acceptability of the WeChat-PBL teaching mode for postgraduates majoring in hematology. The new PBL mode is time-saving and convenient. Additionally, it provides a suitable platform for sharing the latest information and educational resources. It emphasizes interoperable, interactive, effective, and participatory teaching styles. Despite the limited sample size in our study, our results indicate that WeChat-PBL is applicable to postgraduates majoring in hematology. In the future, further comprehensive, large-scale, and high-quality studies should be conducted to confirm our findings and thus promote the applications of this new teaching mode for hematological postgraduates.

## Abbreviations

PBL: problem-based learning
